# Microgalvanic Corrosion of Mg–Ca and Mg–Al–Ca Alloys in NaCl and Na_2_SO_4_ Solutions

**DOI:** 10.3390/ma14237140

**Published:** 2021-11-24

**Authors:** Peixu Yang, Songbo Ye, Baojing Feng, Jinhui Liu, Sensen Huang, Guonan Liu, Weidong Zhang, Weineng Tang, Shijie Zhu, Shaojun Zhang

**Affiliations:** 1 Henan Province Industrial Technology Research Institute of Resources and Materials, Zhengzhou University, Zhengzhou 450001, China; yangpx@zzu.edu.cn (P.Y.); ye12391266@163.com (S.Y.); 13290904100@163.com (B.F.); dingshi95@126.com (G.L.); zhangwd@zzu.edu.cn (W.Z.); zhangs@zzu.edu.cn (S.Z.); 2 School of Materials Science and Engineering, Zhengzhou University, Zhengzhou 450001, China; zhusj@zzu.edu.cn; 3 Institute of Metal Research, Chinese Academy of Sciences, Shenyang 110016, China; 4 Technology Center, Baosteel Metal Co., Ltd., Shanghai 200940, China; tangweineng@baosteel.com; 5 Henan Key Laboratory of Advanced Magnesium Alloy, Ministry of Education, Zhengzhou 450001, China

**Keywords:** Mg–Ca alloy, intermetallic, microgalvanic corrosion, corrosion resistance

## Abstract

As a kind of potential biomedical material, Mg–Ca alloy has attracted much attention. However, the role of Ca-containing intermetallics in microgalvanic corrosion is still controversial. In 0.6 mol/L NaCl and Na_2_SO_4_ solutions, the microgalvanic corrosion behavior of the second phase and Mg matrix of Mg–Ca and Mg–Al–Ca alloys was examined. It was confirmed that the Mg_2_Ca phase acts as a microanode in microgalvanic corrosion in both NaCl and Na_2_SO_4_ solutions, with the Mg matrix acting as the cathode and the Al_2_Ca phase acting as the microcathode to accelerate corrosion of the adjacent Mg matrix. It was also found that Cl^−^ and SO_4_^2−^ have different sensibilities to microgalvanic corrosion.

## 1. Introduction

Magnesium alloys have attracted special interest in many fields for their light weight, high specific strength, and great biodegradability [1,2]. They have considerable potential for application in transportation, aerospace, electronic technology, and especially biomedicine [3,4,5]. Magnesium alloys have a similar density to human bones and good biocompatibility to be used as human implant materials, which is why they are known as revolutionary metal materials [6,7,8,9,10]. However, the corrosion rate is so fast that many hydrogen bubbles are generated in the human tissue, and the Mg(OH)_2_ surface film formed is loose and porous, meaning it has no protective effect during the soaking process [11]. The poor corrosion resistance prevents wider commercial application of Mg alloys [12,13].

Many alloying elements have been added to Mg alloys to increase corrosion resistance and strength for biomedical applications. Recently, rare earth elements [14,15,16,17] have been used as additional elements. However, it is regrettable that rare earth elements are harmful to the human body and not suitable as human implant materials. A small amount of Al is not harmful to the human body, while Ca is essential for the human body [18,19] and is often used as an added element to reduce the grain size [20,21]. Zhu et al. reported a new Mg–Al–Ca alloy in which a majority of deformable Al_2_Ca precipitates were formed. This alloy demonstrated one of the highest combinations of tensile elongation and work hardening capacity among existing Mg alloys [22].

With the increase in Ca content in Mg–Al alloys, the precipitation phase changes from Mg_17_Al_12_ to the Al_2_Ca and Mg_2_Ca phases [23]. The electrochemical property of these intermetallics is worthy of attention. It is well known that the Mg_17_Al_12_ phase is nobler than the Mg matrix and acts as a cathode to accelerate dissolution of the Mg matrix [24]. Ca is a more active element than Mg, and the electrochemical effect of Ca-containing intermetallics differ from that of intermetallics containing Al. Esmaily [25] and Südholz [26] claimed the Mg_2_Ca phase is unique as it possesses a more active corrosion potential than the Mg matrix of all Mg-based intermetallics. Yang [20] added Ca to Mg–Al–Mn alloy and found that Ca-containing intermetallics were initially corroded instead of the Mg matrix. On the other hand, Wu [23] added Al to Mg–Ca alloy and found that Ca-containing intermetallics acted as cathodes and barriers to corrosion. The corrosion behavior of Ca-containing intermetallics in microgalvanic corrosion is still debatable.

Due to the potential application of magnesium–calcium alloys as biomaterials, their corrosion behavior is mostly studied in simulated body fluids [27,28]. However, the corrosion behavior of Mg–Al alloys reported thus far has mostly focused on NaCl solution [29]. In addition, there has been limited research on corrosion of Mg–Al–Ca alloys, and all the known studies were carried out in NaCl solution [30]. The human body fluid contains many ions, such as chloride, phosphate, carbonate, sulfate, etc. [10,11]. According to previous results, Cl^−^ is much more aggressive than SO_4_^2−^ to Mg alloys [31]. The present authors recently found that WE43 (Mg–Y–Gd–Nd–Zr) alloy shows worse corrosion resistance in Na_2_SO_4_ than in NaCl solution [4]. Meanwhile, the microgalvanic corrosion behavior of WE43 alloy in Na_2_SO_4_ solution, where the second phase acts as anode and dissolves preferentially, is poles apart from that in NaCl solution, where the second phase acts as a cathode. It is worth investigating whether Ca-containing intermetallics have different microgalvanic behavior in NaCl and Na_2_SO_4_ solutions.

The present work aimed to study the role of Ca-containing intermetallics in microgalvanic corrosion in NaCl and Na_2_SO_4_ solutions and the corrosion resistance of Mg–Ca and Mg–Al–Ca alloys in the two solutions.

## 2. Experimental Procedure

### 2.1. Materials Preparation

Mg–Ca and Mg–Al–Ca alloys were supplied by YinGuang Magnesium Industry Co., Ltd., Wenxi, China. The nominal composition is listed in Table 1. The ingot casting was cut into 10 mm × 10 mm × 10 mm sample for the immersion test, followed by being ground, polished, degreased, and dried. The specimens connected to the copper wire and sealed with resin were used for electrochemical tests with an exposed area of 1 cm^2^. Subsequently, the working surface was mechanically ground with SiC paper to P2000 grit, cleaned with deionized water and alcohol, and finally dried by flowing cold air.

### 2.2. Microstructure Characterization

The microstructure of the Mg–Ca and Mg–Al–Ca alloys was observed by optical microscopy (OM) and scanning electron microscope (SEM; American FEI Quanta 250 FEG). The phase morphology of the alloys was detected by t backscattered electron (BSE).

### 2.3. Immersion Measurements

#### 2.3.1. Immersion Observation Tests

At 25 ± 0.5 °C, the sample size of 10 × 10 × 10 mm was exposed to 0.6 mol/L NaCl solution and 0.6 mol/L Na_2_SO_4_ solution for 2 min, 5 min, 30 min, 2 h, 5 h, and 24 h for corrosion morphology observation. All specimens were washed with deionized water after immersion, degreased with ethanol, and dried in cold air flow. At room temperature, the specimens were soaked in a chromic acid solution containing 180 g/L CrO_3_ for 5 min to remove the corrosion products. SEM–BSE and EDS were used to examine the corrosion surface after removing corrosion products as well as the cross-sectional morphology of the corroded samples.

#### 2.3.2. Hydrogen Evolution and Weight Loss Test

All the tests were performed in 0.6 mol/L NaCl and 0.6 mol/L Na_2_SO_4_ solutions at 25 ± 0.5 °C. The funnel collected all the hydrogen bubbles over a specimen with the dimension of 30 mm × 30 mm × 10 mm in a beaker containing 2 L of testing solution. The hydrogen evolution was recorded every five minutes for a total of three hours. The weight loss test was carried out using a digital balance (JA3003) before and after soaking with the corrosion products removed by chromic acid.

#### 2.3.3. Electrochemical Measurements

All the experiments were carried out in 0.6 mol/L NaCl and 0.6 mol/L Na_2_SO_4_ solutions using a CHI660D electrochemical workstation. At 25 ± 0.5 °C, a typical three-electrode setup was used with a Pt mesh as the counter electrode, an R232 saturated calomel electrode (SCE) as the reference electrode, and the samples as the working electrode.

EIS tests were performed at open-circuit potential over a frequency range from 100 kHz to 10 mHz, and the sinusoid potential perturbation had an amplitude of 5 mV. The EIS spectra were fitted using the ZSimpWin 3.20 software.

After the EIS test, the cathodic polarization curves were carried out from 0 to −0.3 V vs. the OCP. All the electrochemical tests were replicated more than three times to ensure accuracy of the results. The anodic polarization curves were started at the OCP value and terminated at a final current density of 10 mA cm^−2^. The scan rate was 0.5 mV s^−1^.

## 3. Results and Discussion

### 3.1. Microstructural Analysis

**Figure 1:**
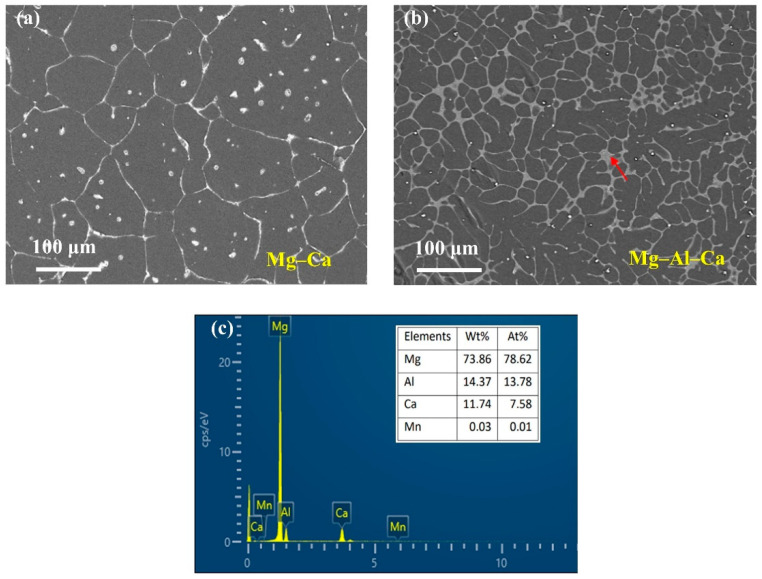
SEM–BSE morphologies of Mg–Ca and Mg–Al–Ca alloys. (**a**) Mg–Ca alloy; (**b**) Mg–Al–Ca alloy; (**c**) EDS results of the second phase (pointed by the red arrow) in Mg–Al–Ca alloy.

**Figure 2:**
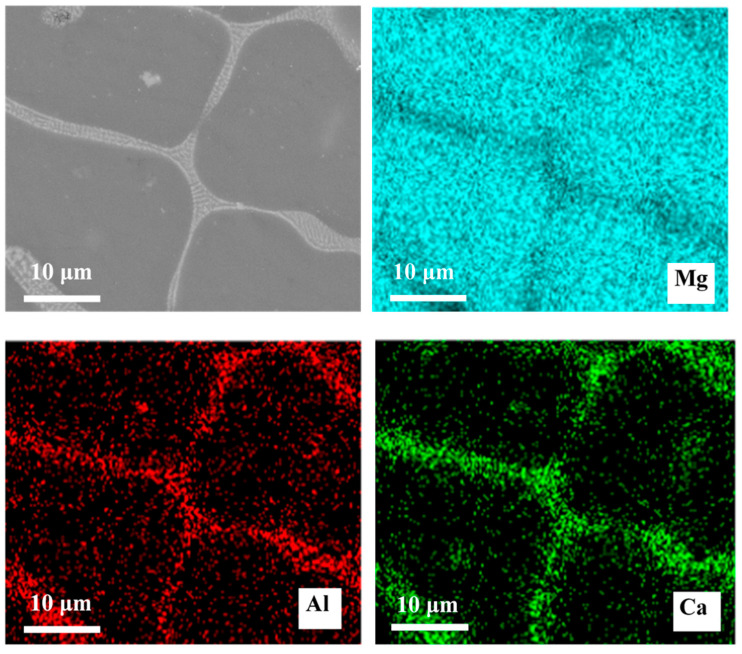
SEM–BSE morphologies of Mg–Al–Ca alloy and EDS mapping of elements.

The SEM micrographs are presented in Figure 1. As can be seen, with the addition of the Al element, the size of the grain became smaller. The number of second phases at the grain boundaries increased, and the second phase morphology changed from a semicontinuous network in Mg–Ca alloys to a continuous network in Mg–Al–Ca alloys. The second phase in the Mg–Ca alloy was the Mg_2_Ca phase, and the types of second phase in Mg–Al–Ca alloys were determined by the mass ratio of Ca/Al [32]. Ninomiya et al. [33] reported that there were two kinds of second phase particles (Mg_2_Ca and Al_2_Ca phase) in Mg–Al–Ca alloys when the mass ratio of Ca/Al was higher than 0.8, while there was only an Al_2_Ca phase particle when the mass ratio of Ca/Al was lower than 0.8. Jiang [34] and Luo [35] confirmed that the second phase containing Ca was (MgAl)_2_Ca with the Ca/Al ratio of 0.50–0.67. The Ca/Al ratio of the Mg–Al–Ca alloy in this work was 0.70. Taking the results of EDS (Figure 1c) and element mapping (Figure 2) into consideration, it was still unclear whether the network second phase in the Mg–Al–Ca alloy was (MgAl)_2_Ca or Al_2_Ca as the detecting area must contain the influence of the adjacent Mg matrix. Luckily, the influence of the Mg matrix could be eliminated after immersion in solution, and it was found that Al_2_Ca was the main component of the network’s second phase, as further discussed in Section 3.2.2. Meanwhile, the SEM–BSE morphology of the Mg–Al–Ca alloy showed bright block phases were distributed along with the network second phase; this was the AlMn phase.

### 3.2. Corrosion Morphology Characterization

#### 3.2.1. Mg–Ca Alloy

**Figure 3:**
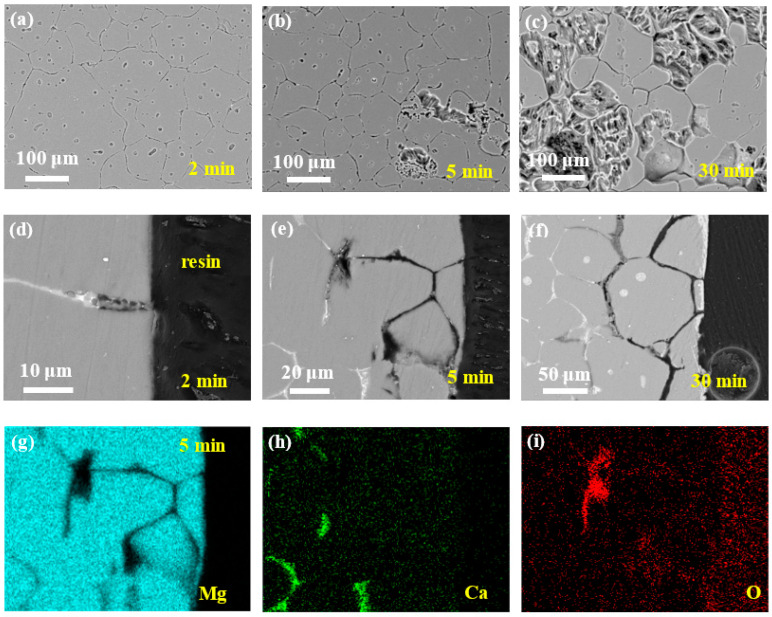
Corrosion morphologies of Mg–Ca alloy after immersion in 0.6 mol/L NaCl solution for different times with corrosion products removed. Surface morphologies after (**a**) 2 min, (**b**) 5 min, and (**c**) 30 min. Cross-section morphologies after (**d**) 2 min, (**e**) 5 min, and (**f**) 30 min. (**g**–**i**) EDS mapping results of 5 min cross-sectional sample in (**e**).

Figure 3 depicts the microgalvanic corrosion behavior of the Mg–Ca alloy’s second phase and Mg matrix in NaCl solution with the corrosion products removed. It was confirmed that the second phase of the Mg–Ca alloy was more active than the Mg matrix and dissolved preferentially in NaCl solution.

After 2 min of immersion, no second phase could be detected on the surface of the Mg–Ca alloy (see Figure 3a), and the depth of dissolution of the second phase was about 10 m from the cross-sectional morphology (see Figure 3d), with the Mg matrix being unaffected. Then, with the increase in the immersion time, the dissolution of the second phase became more and more severe, about 60 and 100 μm of the dissolution depth after immersion for 5 and 30 min, respectively (see Figure 3b–f). Meanwhile, the intensity of the microgalvanic corrosion between the second phase and Mg matrix weakened with the depth of the second phase dissolution increasing, and corrosion of the Mg matrix was triggered. The corrosion of the Mg matrix could be seen on the surface after immersion for 5 min (see Figure 3b), which extended to most of the surface after 30 min (see Figure 3c).

The mapping results of elements Mg, Ca, and O of the sample after immersion for 5 min (Figure 3e) confirmed the dissolution of the second phase. The hole left by the dissolution of the second phase was filled with epoxy resin; thus, the missing Mg and Ca in this area was distinct (see Figure 3g,h). The bottom of the dissolution showed enrichment of O, which were oxides or hydroxides that were not removed by the chromic acid. However, Mg and Ca deficiency was observed in this area, which was unexpected. The oxides or hydroxides should be CaO/Ca(OH)_2_ or MgO/Mg(OH)_2_; thus, there should be the existence of Ca and Mg in this area in the mapping results. Perhaps the color in this area was weak but not void. This phenomenon requires further study.

**Figure 4:**
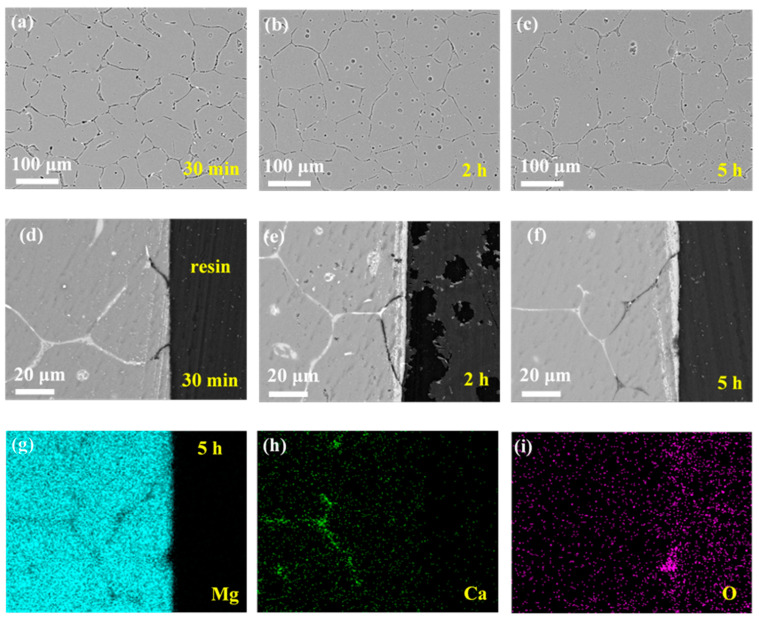
Corrosion morphologies of Mg–Ca alloy after immersion in 0.6 mol/L Na_2_SO_4_ solutions for different times with corrosion products removed. Surface morphologies after (**a**) 30 min, (**b**) 2 h, and (**c**) 5 h. Cross-section morphologies after (**d**) 30 min, (**e**) 2 h, and (**f**) 5 h. (**g**–**i**) EDS mapping results of 5 h cross-sectional sample in (**f**).

Figure 4 shows the microgalvanic corrosion behavior of the Mg–Ca alloy in Na_2_SO_4_ solution. The second phase still served as the anode and dissolved preferentially. However, it was found that the severity of the Mg–Ca alloy corrosion in Na_2_SO_4_ solution was much weaker than that in NaCl solution, meaning the corrosion resistance of the Mg–Ca alloy in Na_2_SO_4_ solution was much better than that in the NaCl solution. After immersion for 30 min and 2 h, there was no existence of second phase on the surface of the Mg–Ca alloy (see Figure 4a,b). The depth of the dissolution of the second phase can be seen in Figure 4d,e, with the Mg matrix unattacked. After immersion for 5 h, the depth of the dissolution of the second phase increased, while small pitting corrosion of the Mg matrix emerged (see Figure 4c,f). This indicated corrosion of the Mg matrix due to dissolution of the second phase and weak microgalvanic corrosion, just like the corrosion phenomenon in NaCl solution. The mapping results of elements Mg, Ca, and O of the sample after immersion for 5 h (Figure 4f), as shown in Figure 4f,h,i, also confirmed the dissolution of the second phase.

The corrosion behavior of the Mg–Ca alloy in NaCl and Na_2_SO_4_ solutions was similar, and the corrosion process of the Mg–Ca alloy in both NaCl and Na_2_SO_4_ solutions could be divided into two stages. First, this step was dominated by microgalvanic corrosion between the second phase and the Mg matrix, with the second phase acting as anode and dissolving preferentially with the Mg matrix protected. Second, with the depth of the second phase dissolution increasing, the microgalvanic corrosion on the surface of the Mg–Ca alloy weakened, and corrosion of the Mg matrix occurred; thus, in this stage, microgalvanic corrosion and dissolution of the Mg matrix took place simultaneously.

#### 3.2.2. Mg–Al–Ca Alloy

**Figure 5:**
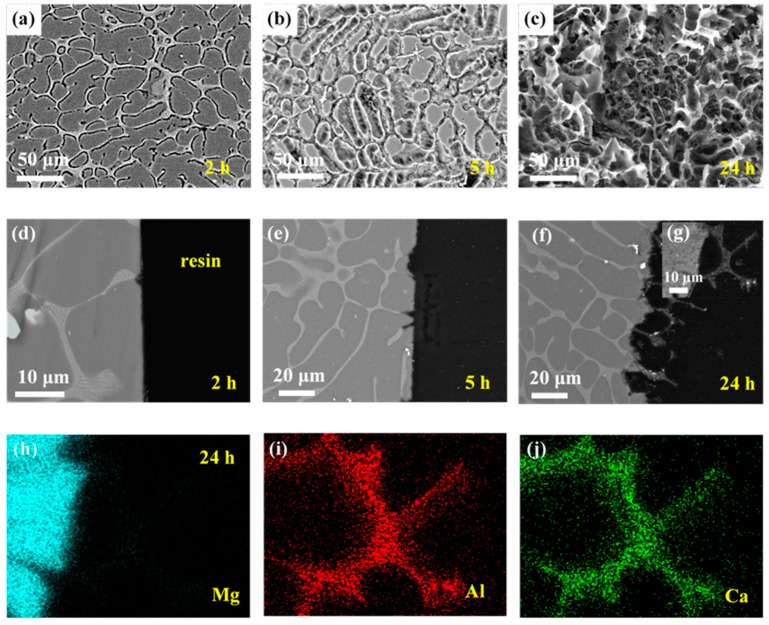
Corrosion morphologies of Mg–Al–Ca alloy after immersion in 0.6 mol/L NaCl solution for different times with corrosion products removed. Surface morphologies after (**a**) 2 h, (**b**) 5 h, and (**c**) 24 h. Cross-section morphologies after (**d**) 2 h, (**e**) 5 h, and (**f**,**g**) 24 h. (**h**–**j**) EDS mapping results of 24 h cross-sectional sample in (**g**).

The corrosion morphologies of the Mg–Al–Ca alloy in NaCl solution with corrosion products removed are shown in Figure 5. Contrary to the microgalvanic corrosion behavior of the Mg–Ca alloy, the second phase in the Mg–Al–Ca alloy acted as the cathode and accelerated corrosion of the Mg matrix. In NaCl solution, firstly, the Mg matrix adjacent to the second phase corroded, as seen in Figure 5a. The origination of microgalvanic corrosion can be appreciably observed in the cross-sectional morphology in Figure 5d. After immersion for 5 h, microgalvanic corrosion proceeded, the extension of the corrosion area was no longer limited adjacent to the second phase anymore, and the island of the intact Mg matrix was distributed on the surface (see Figure 5b,e). Then, after immersion for 24 h, the whole surface suffered severe corrosion with the remnant of the network second phase (see Figure 5c). In the cross-sectional morphology in Figure 5f,g, dissolution of the Mg matrix and the residual network second phase can be appreciably observed.

The mapping results of elements Mg, Al, and Ca of the sample after immersion for 24 h (Figure 5g), as shown in Figure 5h–j, confirmed the network second phase acting as the cathode during microgalvanic corrosion. Meanwhile, the results of mapping identified the component of the second phase of the Mg–Al–Ca alloy. The enrichment of Al and Ca in the second phase is shown in Figure 5i,j; however, few Mg element existed in this area. Thus, the second phase in the Mg–Al–Ca alloy was Al_2_Ca, not (MgAl)_2_Ca.

**Figure 6:**
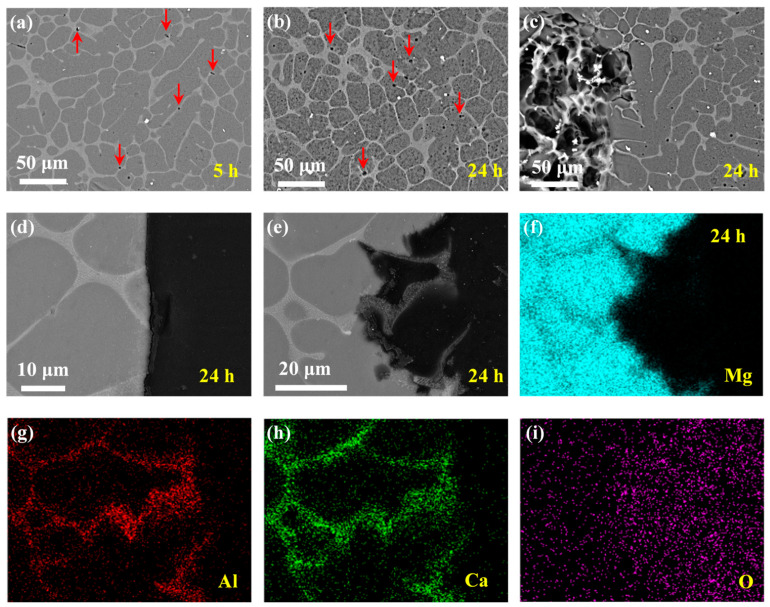
Corrosion morphologies of Mg–Al–Ca alloy after immersion in 0.6 mol/L Na_2_SO_4_ solutions for different times with corrosion products removed. Surface morphologies after (**a**) 5 h and (**b**,**c**) 24 h. Cross-sectional morphologies (**d**) corresponding to (**b**,**e**) corresponding to (**c**). (**f**–**i**) EDS mapping results of 24 h cross-sectional sample in (**e**).

The corrosion morphologies of the Mg–Al–Ca alloy in Na_2_SO_4_ solution with corrosion products removed are shown in Figure 6. It was observed that the corrosion rate of the Mg–Al–Ca alloy in Na_2_SO_4_ solution was much slower than that in NaCl solution. The slight corrosion of the surface morphology after immersion for 5 h is shown in Figure 6a. Only some holes (indicated by the red arrows) could be seen on the surface resulting from the falling of the AlMn phase. Then, with the immersion time increasing, the morphology in Figure 6b dominated the surface after immersion for 24 h (the corresponding cross-section morphology is shown in Figure 6d). Apart from the falling of the AlMn phase, small pitting corrosion or uniform corrosion of Mg matrix occurred on the surface, not microgalvanic corrosion between the Al_2_Ca phase and Mg matrix like the corrosion behavior of the Mg–Al–Ca alloy in NaCl solution. Occasionally, there were one or two corrosion pits on the surface (see Figure 6c; corresponding cross-section morphology is shown in Figure 6e). In this area, obvious microgalvanic corrosion between the Al_2_Ca phase and Mg matrix was present, with the Al_2_Ca phase acting as the cathode. The mapping results of elements Mg, Al, Ca, and O are shown in Figure 6f–i, respectively. The corrosion pit could be attributed to the distribution of the AlMn phase and impurities.

The corrosion behavior of the Mg–Al–Ca alloy in NaCl solution was significantly different from that in Na_2_SO_4_ solution. In NaCl solution, the microgalvanic corrosion between the Al_2_Ca phase and Mg matrix of the Mg–Al–Ca alloy dominated the whole immersion process, with the Al_2_Ca phase acting as cathode. However, in the Na_2_SO_4_ solution, uniform corrosion of the Mg matrix was the main phenomenon. In other words, NaCl solution was much more sensitive to the difference between phases in the Mg–Al–Ca alloy than Na_2_SO_4_ solution.

### 3.3. Hydrogen Evolution and Weight Loss Test

**Figure 7:**
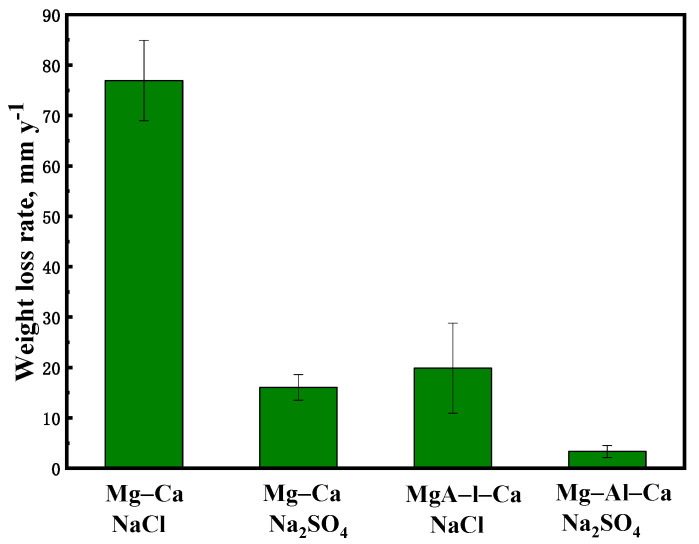
The weight loss rate of Mg–Ca and Mg–Al–Ca alloys after soaking in 0.6 mol/L NaCl and Na_2_SO_4_ solutions for 2 d.

The weight loss rates of the Mg–Ca and Mg–Al–Ca alloys immersed in 0.6 mol/L NaCl solution and 0.6 mol/L Na_2_SO_4_ solution for two days are shown in Figure 7. The weight loss rate of the Mg–Ca alloy in NaCl solution (76.92 mm/y) was about 5 times faster than that in Na_2_SO_4_ solution (16.03 mm/y), and the weight loss rate of the Mg–Al–Ca alloy in NaCl solution (19.86 mm/y) was about 6 times as fast as that in Na_2_SO_4_ solution (3.33 mm/y). Both the Mg–Ca and Mg–Al–Ca alloys showed faster corrosion rates in NaCl solution than in Na_2_SO_4_ solution, which coincided with the corrosion morphologies. This phenomenon is attributed to Cl^−^ being more corrosive than SO_4_^2−^ [31]. The WE43 alloy in these two solutions showed a different result, which is interesting [4].

On the other hand, the corrosion resistance of the alloy became better with the addition of Al. In NaCl solution, the weight loss rate of the Mg–Ca alloy was about 4 times faster than that of the Mg–Al–Ca alloy. In Na_2_SO_4_ solution, the weight loss rate of the Mg–Ca alloy was about 5 times as fast as that of the Mg–Al–Ca alloy.

**Figure 8:**
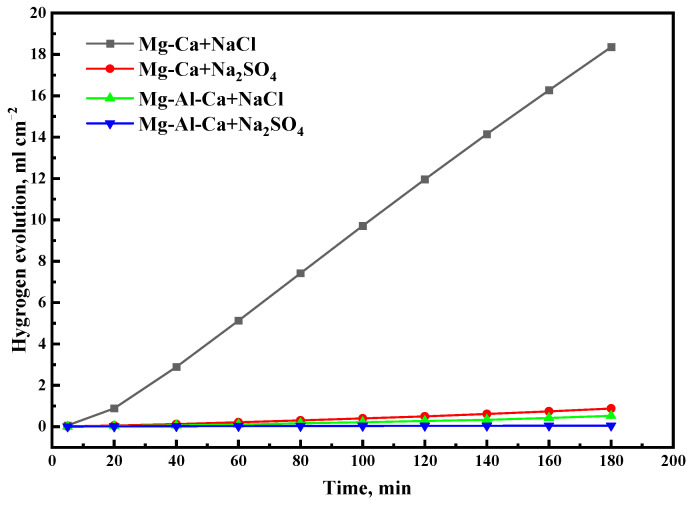
Hydrogen evolution of Mg–Ca and Mg–Al–Ca alloys after immersion in 0.6 M NaCl and Na_2_SO_4_ solutions for 3 h.

The hydrogen evolution of the two alloys immersed in 0.6 mol/L NaCl and Na_2_SO_4_ solutions for 3 h are shown in Figure 8. The hydrogen evolution of the Mg–Ca alloy was faster than that of the Mg–Al–Ca alloy, and it was faster in NaCl solution than in Na_2_SO_4_ solution. This result was consistent with the weight loss rate.

### 3.4. Electrochemical Measurements

#### 3.4.1. EIS Analysis

**Figure 9:**
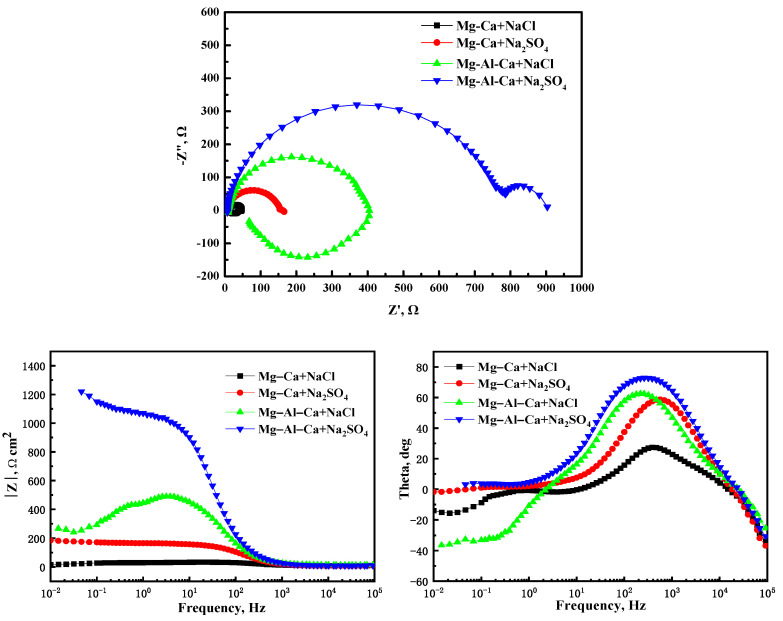
The EIS plots of Mg–Ca alloy and Mg–Al–Ca alloys in 0.6 mol/L NaCl and Na_2_SO_4_ solutions.

Figure 9 shows the EIS plots of the Mg–Ca and Mg–Al–Ca alloys soaked in 0.6 mol/L NaCl and Na_2_SO_4_ solutions. The plots of the alloys in Na_2_SO_4_ solution consisted of a high-frequency and a medium-frequency capacitance loop, while the plots of the alloys in NaCl solution had a low-frequency inductance loop. The high-frequency capacitance loop can be associated with the electric double layer at the interface of the Mg substrate and electrolyte. The medium-frequency capacitance loop should be related to the surface film. The low-frequency loop can be attributed to the initiation of localized corrosion.

**Figure 10:**
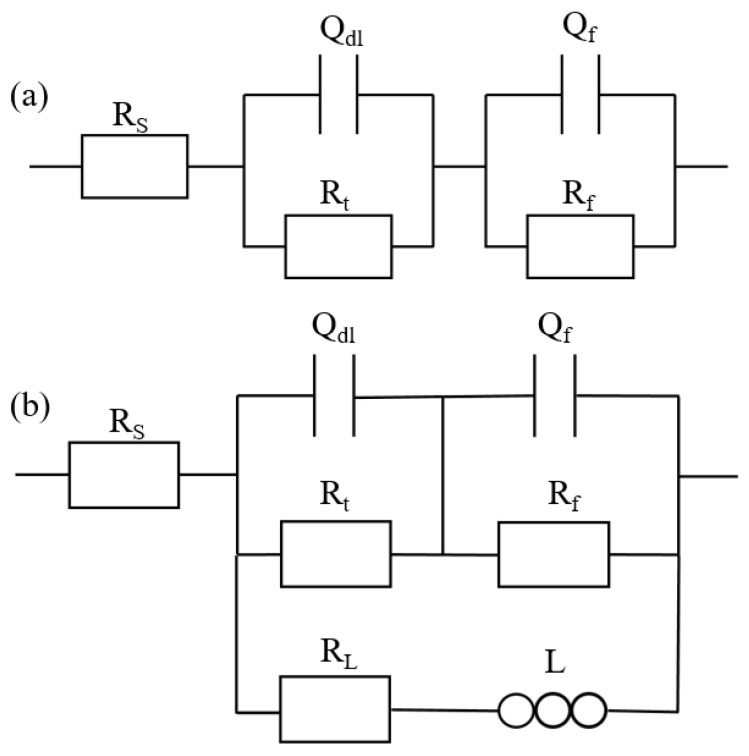
Equivalent circuit diagram of Mg–Ca and Mg–Al–Ca alloys in (**a**) Na_2_SO_4_ solution and (**b**) NaCl solution.

The equivalent circuit diagram is shown in Figure 10, and the fitting results are given in Table 2. In Figure 10, R_s_ is the solution resistance, R_t_ is the charge transfer resistance, Q_dl_ is the capacitance of the electric double layer, R_t_ and Q_dl_ together indicate the first capacitance loop in high-frequency, and R_f_ and Q_f_ are the surface film resistance and capacitance, respectively. In addition, L is the inductance of the electrochemical reactions at the film–substrate interface, and the R_L_ is the resistance of the inductance [31].

The capacitive diameter of the Mg–Ca and Mg–Al–Ca alloys in Na_2_SO_4_ solution was much larger than that in NaCl solution. Meanwhile, the Nyquist plots of the two alloys in NaCl solution showed a distinctive low-frequency inductance loop. These results indicate that the corrosion resistance of two alloys in Na_2_SO_4_ solution is better than in NaCl solution, which coincides with the results of corrosion morphologies, weight loss, and hydrogen evolution.

#### 3.4.2. Potential Dynamic Polarization Curves

**Figure 11:**
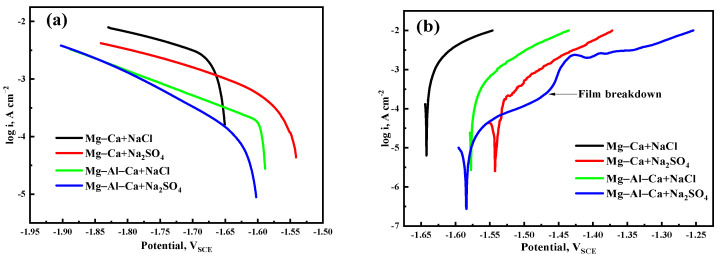
Polarization curves of Mg–Ca and Mg–Al–Ca alloys in 0.6 mol/L NaCl and Na_2_SO_4_ solutions: (**a**) cathodic curves and (**b**) anodic curves. The polarization test of Mg–Ca alloy immersed in NaCl solution, Mg–Ca alloy immersed in Na_2_SO_4_ solution, Mg–Al–Ca alloy immersed in NaCl solution, and Mg–Al–Ca alloy immersed in Na_2_SO_4_ solution started from −1.6512, −1.5424, −1.5836, and −1.6014 V_SCE_, respectively.

The cathodic and anodic polarization curves of the Mg–Ca and Mg–Al–Ca alloys immersed in 0.6 mol/L NaCl and Na_2_SO_4_ solutions are shown in Figure 11. Before the polarization test, the open-circuit potential was obtained, and the open-circuit potentials of Mg–Ca alloys immersed in NaCl solution, Mg–Ca alloys immersed in Na_2_SO_4_ solution, Mg–Al–Ca alloys immersed in NaCl solution, and Mg–Al–Ca alloys immersed in Na_2_SO_4_ solutions were −1.6422, −1.5424, −1.5776, and −1.584, respectively.

It is generally believed that the cathode of the polarization curve represents a hydrogen evolution reaction, and the anode is related to the anodic dissolution of the magnesium alloy. The fitting results of the cathodic polarization curves in Figure 11 are listed in Table 3. The I_corr_ values of the two alloys in Na_2_SO_4_ solution were much smaller than those in NaCl solution. Meanwhile, the anodic polarization curve of the Mg–Al–Ca alloy in Na_2_SO_4_ solution showed passivation of the surface film, which terminated at the turning point of film breakdown. However, the other three curves were always active dissolution. This indicates that the surface film of Mg–Al–Ca alloy is more protective than that of the Mg–Ca alloy, and Cl^−^ is more corrosive than SO_4_^2−^ to the surface film.

## 4. Conclusions

1. The second phase (Mg_2_Ca) of the Mg–Ca alloy serves as the microanode in microgalvanic corrosion with the Mg matrix in NaCl and Na_2_SO_4_ solutions. However, the second phase (Al_2_Ca) of the Mg–Al–Ca alloy serves as the microcathode in microgalvanic corrosion in NaCl and Na_2_SO_4_ solutions.

2. The addition of Al can significantly improve the corrosion resistance of the Mg–Ca alloy in both NaCl and Na_2_SO_4_ solutions.

3. Cl^−^ ion is more sensitive to microgalvanic corrosion between different phases and more corrosive to the surface film than SO_4_^2−^ ion.

## Figures and Tables

**Table 1:** The nominal composition of Mg–Ca and Mg–Al–Ca alloys (wt %).

Alloys	Al	Ca	Mn	Zn	Mg
Mg–Ca	-	3.01	0.34	0.011	Bal.
Mg–Al–Ca	3.64	2.56	0.35	0.013	Bal.

**Table 2:** EIS fitting results.

State	R_S_	Q_dl_	n_dl_	R_t_	Q_f_	n_f_	R_f_	R_L_	L
Mg–Ca + NaCl	9.84	2.19 × 10^−5^	0.99	31.26	4.11 × 10^−5^	0.99	2.296	7.462	80.77
Mg–Ca + Na_2_SO_4_	7.83	9.67 × 10^−6^	0.99	104.8	7.22 × 10^−4^	0.56	45.09	/	/
Mg–Al–Ca + NaCl	9.70	8.89 × 10^−6^	0.99	366.3	3.28 × 10^−5^	0.96	5.543	85.28	200.4
Mg–Al–Ca + Na_2_SO_4_	7.47	6.41 × 10^−6^	0.99	633.4	3.23 × 10^−4^	0.65	293.8	/	/

**Table 3:** Fitting results of the cathodic polarization curves.

State	E_corr_ (V_SCE_)	I_corr_ (μA cm^−2^)	B_c_ (mV Decade^−1^)
Mg–Ca + NaCl	−1.66	841.8	62.57
Mg–Ca + Na_2_SO_4_	−1.53	129.9	58.61
Mg–Al–Ca + NaCl	−1.58	98.08	40.02
Mg–Al–Ca + Na_2_SO_4_	−1.60	30.37	59.24

## Data Availability

All data used to support the findings of this study are included in the article.
